# Epigenome-wide association study of biomarkers of liver function identifies albumin-associated DNA methylation sites among male veterans with HIV

**DOI:** 10.3389/fgene.2022.1020871

**Published:** 2022-10-11

**Authors:** Boghuma K. Titanji, Mitch Lee, Zeyuan Wang, Junyu Chen, Qin Hui, Vincent Lo Re III, Kaku So-Armah, Amy C. Justice, Ke Xu, Matthew Freiberg, Marta Gwinn, Vincent C. Marconi, Yan V. Sun

**Affiliations:** ^1^ Division of Infectious Disease, Emory School of Medicine, Atlanta, GA, United States; ^2^ Department of Epidemiology, Rollins School of Public Health, Emory University, Atlanta, GA, United States; ^3^ Division of Infectious Diseases Department of Medicine and Center for Clinical Epidemiology and Biostatistics Perelman School of Medicine University of Pennsylvania, Philadelphia, PA, United States; ^4^ Boston University Medical School, Boston, MA, United States; ^5^ Connecticut Veteran Health System, West Haven, CT, United States; ^6^ Yale University School of Medicine, New Haven, CT, United States; ^7^ Cardiovascular Medicine Division and Tennessee Valley Healthcare System, Vanderbilt University Medical Center, Nashville, TN, United States; ^8^ Centers for Disease Control and Prevention, Atlanta, GA, United States; ^9^ Atlanta Veterans Affairs Health Care System, Decatur, GA, United States; ^10^ Hubert Department of Global Health, Rollins School of Public Health, Atlanta, GA, United States; ^11^ Emory Vaccine Center, Yerkes National Primate Research Center, Emory University, Atlanta, GA, United States

**Keywords:** EWAS, liver disease, HIV, biomarkers, albumin (ALB)

## Abstract

**Background**: Liver disease (LD) is an important cause of morbidity and mortality for people with HIV (PWH). The molecular factors linked with LD in PWH are varied and incompletely characterized. We performed an epigenome-wide association study (EWAS) to identify associations between DNA methylation (DNAm) and biomarkers of liver function—aspartate transaminase, alanine transaminase, albumin, total bilirubin, platelet count, FIB-4 score, and APRI score—in male United States veterans with HIV.

**Methods:** Blood samples and clinical data were obtained from 960 HIV-infected male PWH from the Veterans Aging Cohort Study. DNAm was assessed using the Illumina 450K or the EPIC 850K array in two mutually exclusive subsets. We performed a meta-analysis for each DNAm site measured by either platform. We also examined the associations between four measures of DNAm age acceleration (AA) and liver biomarkers.

**Results:** Nine DNAm sites were positively associated with serum albumin in the meta-analysis of the EPIC and 450K EWAS after correcting for multiple testing. Four DNAm sites (cg16936953, cg18942579, cg01409343, and cg12054453), annotated within the *TMEM49* and four of the remaining five sites (cg18181703, cg03546163, cg20995564, and cg23966214) annotated to *SOCS3*, *FKBP5*, *ZEB2*, and *SAMD14* genes, respectively. The DNAm site, cg12992827, was not annotated to any known coding sequence. No significant associations were detected for the other six liver biomarkers. Higher PhenoAA was significantly associated with lower level of serum albumin (*β* = -0.007, *p*-value = 8.6 × 10^–4^, CI: -0.011116, -0.002884).

**Conclusion:** We identified epigenetic associations of both individual DNAm sites and DNAm AA with liver function through serum albumin in men with HIV. Further replication analyses in independent cohorts are warranted to confirm the epigenetic mechanisms underlying liver function and LD in PWH.

## Graphical Abstract

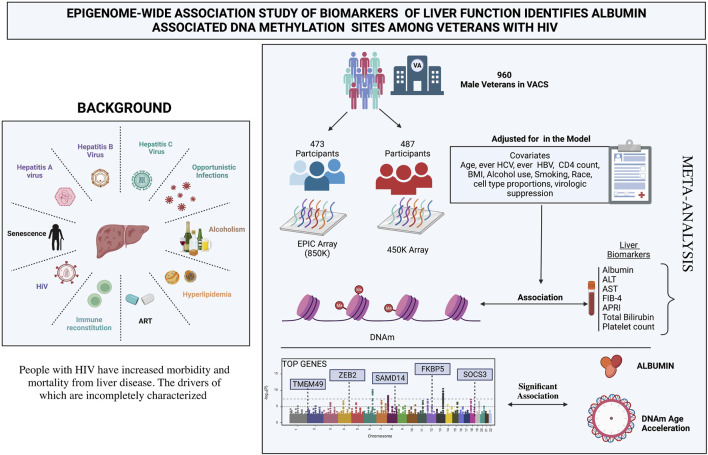

## Introduction

The widespread availability and use of effective combination antiretroviral therapy to treat HIV has transformed the outlook for people with HIV (PWH), many of whom now live longer and are facing the challenges of aging with HIV. The burden of non-AIDS related chronic disease (NACD) is increasingly recognized in this patient population as an urgent concern that needs to be addressed to improve care for PWH.

Liver disease (LD) accounts for an estimated 13%–18% all-cause mortality in PWH, and PWH have a high prevalence of traditional causes of LD (Palella et al., 2006; Smith et al., 2010; Smith et al., 2014; Kaspar and Sterling, 2017). Co-infection with hepatitis B and C viruses is estimated between 5%–30% based on cohort studies (Konopnicki et al., 2005; Shepard et al., 2005), and up to 30%–40% of PWH have signs of non-alcoholic fatty liver disease (NAFLD) (Lemoine et al., 2012). Poorly controlled HIV-infection itself is an independent risk factor for liver fibrosis (Kim et al., 2016a), which is the most common outcome of chronic liver injury. Even in individuals with well-controlled HIV (undetectable HIV RNA) and immunologic recovery (CD4^+^ cell counts > 500 cells/mm^3^), oxidative stress, mitochondrial injury, toxic metabolite accumulation, gut microbial translocation, systemic inflammation, senescence, and nodular regenerative hyperplasia [reviewed in (Kim et al., 2016a)] contribute to liver injury and fibrosis. Given all of the factors contributing to LD among PWH, there is a need to improve prevention, risk prediction, and treatment of LD in PWH.

Progress in elucidating the biological mechanisms underlying LD has been challenging due to the complexity of the multiple roles played by the liver in homeostasis and metabolic functions. Epigenome-wide association study (EWAS) have the potential to identify novel mechanisms of LD and generate new hypotheses that may provide insights into prevention and treatment of LD. Previous studies on the role of DNA methylation (DNAm) in LD have focused on non-alcoholic fatty LD (NAFLD), since determinants of NAFLD are not as easily identifiable as LD resulting from e.g., alcoholism or viral hepatitis [reviewed in (Zhang et al., 2021)]. Studies on the association between alcoholic liver disease and DNAm have identified genes which may be associated with the incidence and progression of hepatocellular carcinoma (HCC) (French, 2013; Varela-Rey et al., 2013; Zakhari, 2013; Rosen et al., 2018). Other EWAS have uncovered DNAm sites and genes associated with the development of HCC in patients with chronic hepatitis B infections (Su et al., 2007; Nishida et al., 2008; Zhao et al., 2014). PWH are significantly underrepresented in these studies. Moreover, most studies have focused on LD as an overall clinical diagnosis and have not assessed potential associations between DNAm and specific metabolic functions of the liver. Biomarkers are useful surrogates of the liver’s metabolic functions and may reflect its capacity to perform specific functions. The absence of data on the relationship between DNAm and specific markers of liver function, particularly among PWH, represents an important gap in the literature which we hope the present study will begin to fill.

DNAm age acceleration (AA) is a novel biomarker of biological aging, and predicts age-related disease outcomes and mortality (Bell et al., 2019). Several studies have also shown that PWH exhibit elevated DNAm AA above the average level exhibited by non-infected people of equivalent chronological age. (Horvath and Levine, 2015; Rickabaugh et al., 2015). These observations suggest that DNAm AA may partially explain the early onset of chronic comorbid conditions among PWH (Levine et al., 2015a). Relative acceleration of DNAm age from blood-based assays is often measured by four metrics: intrinsic epigenetic age acceleration (IEAA) (Levine et al., 2015a; Horvath and Levine, 2015), extrinsic epigenetic age acceleration (EEAA) (Hannum et al., 2013; Levine et al., 2015b), phenotypic age acceleration (PhenoAA) (Levine et al., 2018), and Grim age acceleration (GrimAA) (Lu et al., 2019).

The present study included available markers of liver function from the Veterans Aging Cohort Study (VACS): aspartate transaminase (AST), alanine transaminase (ALT), serum albumin, total bilirubin, platelet count, fibrosis-4 (FIB-4) score, and the AST-to-platelet-ratio index (APRI) score. We conducted epigenetic association analyses between individual DNAm sites, measures of DNAm AA (IEAA, EEAA, PhenoAA and GrimAA) and these seven biomarkers of liver function in a cohort of male veterans with HIV infection in the United States.

## Methods

### Study population

A total of 960 male veterans with HIV at the time of blood collection were included from the VACS, having both phenotypic and epigenetic data available from previous studies (Justice et al., 2006; Chen et al., 2020). Participants with cancer at the time of blood collection were excluded because the cancer treatment and disease can influence liver function and DNAm which is beyond the scope of the present study. The VACS consists of electronic medical record data from patients with HIV receiving care at Veterans Health Administration (VA) medical facilities across the United States Each PWH is matched on age, sex, race/ethnicity, and site to two persons without HIV. Data include hospital and outpatient diagnoses (recorded using International Classification of Diseases, Ninth Revision [ICD-9] and Tenth [ICD-10] codes), procedures (recorded using Current Procedural Terminology [CPT] codes), laboratory results, and pharmacy data (Justice et al., 2006). Information on age, race, smoking status, body mass index (BMI), diabetes status (glucose level ≥ 200 mg/dl on 2 separate occasions or glucose level ≥ 200 mg/dl on 1 occasion plus treatment with an oral hypoglycemic or insulin for ≥ 30 days) (Mathur et al., 2019), ever infection with HBV (defined as HBV surface antigen positive, acute resolved HBV or ICD-9 code for HBV diagnosis), ever infection with HCV (defined as HCV antibody positive regardless of HCV RNA, ICD-9 code for HCV diagnosis, or HCV genotype), and antiretroviral (ART) use at time of blood draw were obtained from VA’s clinical data warehouse. Alcohol use status was obtained from the VACS survey (Justice et al., 2006). At the visit of blood draw, AST, ALT, serum albumin, total bilirubin, platelet count, CD4^+^ T-cell count, and plasma HIV-1 RNA viral load were measured for each patient using approved clinical assays from certified clinical laboratories. Participants were categorized as HIV suppressed if their HIV RNA was < 200 copies/mm^3^ or as unsuppressed VL otherwise. A fibrosis-4 (FIB-4) score and an AST-to-platelet ratio index (APRI) score were calculated for each participant following the standard equations (Wai et al., 2003; Sterling et al., 2006). All participants provided written consent for the use of their data.

### DNAm data generation, processing, and quality control

The genome-wide DNAm profiles were measured using genome DNA samples extract from whole blood. Genomic DNA extraction, epityping, data processing, and quality control were performed as previously described. (Chen et al., 2020). Genome-wide DNAm levels for 473 of the participants were assessed using the Infinium HumanMethylation450 (450K) array platform (Illumina), while those for the remaining 487 participants were assessed using the Infinium HumanMethylationEPIC (EPIC) array platform (Illumina) at the Yale Center for Genomic Analysis. Quality control procedures were followed as previously described in published studies (Zhang et al., 2017; Solomon et al., 2018; Chen et al., 2020). We compared the characteristics between the 450K and EPIC subsets using t-tests for continuous variables and chi-square tests for categorical variables.

DNAm sites for 412,583 and 846,604 DNAm sites were included in the analyses for the dataset acquired with the 450K and EPIC arrays, respectively. Differences in the proportions of the six main leukocyte cell types present in whole blood (CD4^+^ T cells, CD8^+^ T cells, monocytes, B cells, granulocytes, and natural killer cells) across samples are well described confounders of associations between DNAm in the blood and many phenotypes. To account for this, the proportions of these six cell types for each participant were determined based on the top cell-type-specific DNAm sites in a reference panel of known proportions following the standard algorithm through the *minfi* package in R (Houseman et al., 2012; Moran et al., 2016). The estimated cell type proportions were then controlled for in all EWAS analyses.

The association between each covariate included in the EWAS model—race, smoking status, BMI, diabetes status, hazardous alcohol use (Freiberg et al., 1999), ever HCV infection, ever HBV infection, ART use, CD4 count, viral suppression, and leukocyte cell-type proportions, top ten principal components—and each liver marker was assessed using a linear model controlling for chronological age as in the following model.

### Individual liver marker ∼ covariate + age

Associations between each liver marker and chronological age were also assessed using a univariate model.

### Individual liver marker ∼ age

Regardless of statistical significance, all covariates listed above were included in the final EWAS model to account for the reasonable possibility that they might confound the relationship between DNAm and the liver markers as in the following model.

Individual liver marker ∼ DNAm + age + race +current smoking + BMI + diabetes + hazardous alcohol use + ever infection with HCV + ever infection with HBV + ART use + CD4^+^ T-cell count + VL + leukocyte cell type proportions + top ten principal components.

### Correlations among liver biomarkers

To identify correlations among the biomarkers of liver function included in this study, a Spearman correlation coefficient and corresponding *p*-value were calculated for every pair of selected biomarkers using the R statistical software package. To normalize the distributions of strongly right-skewed biomarkers for this and all subsequent analyses, a natural log transformation was performed on the measured ALT, AST, and calculated FIB-4 and APRI scores.

### Associations of DNAm age acceleration with liver biomarkers

DNAm AA for each participant was measured using the IEAA, EEAA, PhenoAA, and GrimAA metrics as specified by original reporting articles (Chen et al., 2016; Levine et al., 2018; Lu et al., 2019). IEAA is calculated as the residual of a person’s DNAm level in blood cells across a set of DNAm sites proposed by Horvath after regressing DNAm level on chronological age and controlling for proportions of different leukocytes within the sample (Levine et al., 2015a; Horvath and Levine, 2015). EEAA is calculated similarly, except that a different set of DNAm sites proposed by Hanum are used while controlling for proportions of a different set of cell types (Hannum et al., 2013; Levine et al., 2018). PhenoAA, in contrast, is calculated by first estimating phenotypic age (in years) with a linear regression model that uses clinical variables as inputs, then estimating DNAm age (in years) based on methylation levels at a set of DNAm sites proposed by Levine et al. (2018) and finally calculating the residual that results from regressing calculated DNAm age on calculated phenotypic age. GrimAA is calculated in a similar way as PhenoAA but utilizes levels of plasma biomarkers indicative of physiological stress to estimate phenotypic age and a different set of DNAm sites to assess DNAm age (Lu et al., 2019). Each of these measures of DNAm AA perform differently depending on the outcome being evaluated and can complement each other to assess the biological aging process.

The association between DNAm AA, as measured by each of these four metrics, and each of the selected biomarkers of liver function was then assessed by linear regression while controlling for all covariates included in the final EWAS model.

Individual liver marker ∼ DNAm AA + age + race +current smoking + BMI + diabetes + hazardous alcohol use + ever infection with HCV + ever infection with HBV + ART use + CD4^+^ T-cell count + VL + leukocyte cell type proportions + top ten principal components.

### Meta-analysis of EWAS for liver biomarkers

To investigate the association between each biomarker of liver function and individual DNAm sites across the autosomal chromosomes, data from the 450K dataset were analyzed in parallel with data from the EPIC dataset using the same model, and a fixed effect model meta-analysis was conducted using inverse variance weighted effect size method. The final model was adjusted for age, race, current smoking, BMI, diabetes, hazardous alcohol use, ever infection with HCV, ever infection with HBV, ART use, CD4^+^ T-cell count, VL, leukocyte cell type proportions, and the top ten principal components within the group being analyzed. Where necessary for any given combination of DNAm site and biomarker, participants missing values required for the model were excluded from the analysis for that combination.

A total of 385,062 DNAm sites measured by both the 450K, and EPIC arrays were included in the EWAS meta-analysis. For DNAm sites not covered by both platforms, results were obtained only from the cohort profiled by the covering platform and no meta-analysis was conducted. Separate false-discovery rate adjustments (*q <* 0.05) were conducted for each liver biomarker. All analyses were performed in R (v4.0.3).

## Results

### Participant characteristics

After data processing and quality control, the analysis dataset for those profiled using the 450K and EPIC platforms included observations from 473 to 487 individuals, respectively. Characteristics of the two EWAS subsets are summarized in Table 1. All participants were male veterans with HIV who were never diagnosed with cancer and had an average age of 51.2 ± 7.5 years. The chronological age between the 450K and EPIC sub-cohorts was moderately different (*Δ* = 1.1 years, *p*-value = 0.031). The distribution of two sub-cohorts did not differ significantly (*p*-value > 0.05) in race (Black vs. non-Black) or in prevalence of current smoking, diabetes, hazardous alcohol use, ever infection with HBV, ever infection with HCV, or viral load suppression. They also did not differ significantly in their average BMI or CD4^+^ T-cell count. Average biomarker values from the 450K cohort differed significantly from those from the EPIC cohort for ALT (*Δ* = 4.7 units/L, *p*-value = 1.93 × 10^–3^), AST (*Δ* = 6.4 units/L, *p*-value = 7.34 × 10^–5^), serum albumin (*Δ* = -0.11 mg/dl, *p*-value = 2.89 × 10^–4^), FIB-4 score (*Δ* = 0.24, *p*-value = 8.49 × 10^–4^), and APRI score (*Δ* = 0.112, *p*-value = 8.35 × 10^–4^). Total bilirubin and platelet count did not differ significantly between the two sub-cohorts.

**TABLE 1:** Characteristics of participants grouped by platform used for genome-wide DNA methylation.

Characteristic	450K (*N* = 473)	EPIC (*N* = 488)	Overall (*N* = 961)	*p*-value
ALT (units/L)	38.8 (24.9)	34.1 (21.2)	36.4 (23.2)	1.9 × 10^–3^
AST (units/L)	43.4 (28.1)	37.0 (20.1)	40.2 (24.6)	7.3 × 10^–5^
Serum Albumin (mg/dl)	3.84 (0.489)	3.95 (0.457)	3.90 (0.476)	2.9 × 10^–4^
Total Serum Bilirubin (mg/dl)	0.78 (0.485)	0.76 (0.513)	0.77 (0.500)	0.61
Platelet Count [x10 (Zhang et al., 2021)/L]	224 (71.3)	232 (69.4)	228 (70.4)	8.0 × 10^–2^
FIB-4 Score	1.81 (1.22)	1.57 (0.943)	1.69 (1.09)	8.5 × 10^–4^
APRI Score	0.58 (0.602)	0.47 (0.393)	0.52 (0.509)	8.4 × 10^–4^
Age (years)	51.8 (7.53)	50.7 (7.48)	51.2 (7.52)	3.1 × 10^–2^
Race				1.00
Black	402 (85.0%)	391 (80.1%)	793 (82.5%)	
Non-Black	71 (15.0%)	97 (19.9%)	168 (17.5%)	
Smoking Status				1.00
Not current	203 (42.9%)	210 (43.0%)	413 (43.0%)	
Current	270 (57.1%)	278 (57.0%)	548 (57.0%)	
Diabetes Status				1.00
No	390 (82.5%)	406 (83.2%)	796 (82.8%)	
Yes	83 (17.5%)	82 (16.8%)	165 (17.2%)	
BMI	25.4 (4.44)	25.9 (4.43)	25.6 (4.44)	0.14
Missing	8 (1.7%)	9 (1.8%)	17 (1.8%)	
Alcohol Use				1.00
Non-Hazardous	195 (41.2%)	191 (39.1%)	386 (40.2%)	
Hazardous	276 (58.4%)	297 (60.9%)	573 (59.6%)	
Ever Hepatitis C Infection				1.00
No	241 (51.0%)	312 (63.9%)	553 (57.5%)	
Yes	232 (49.0%)	176 (36.1%)	408 (42.5%)	
Ever Hepatitis B Infection				1.00
No	399 (84.4%)	430 (88.1%)	829 (86.3%)	
Yes	52 (11.0%)	40 (8.2%)	92 (9.6%)	
CD4^+^ Cell Count	411 (252)	442 (263)	427 (258)	6.4 × 10^–2^
HIV viral Load				1.00
Suppressed	196 (41.4%)	204 (41.8%)	400 (41.6%)	
Unsuppressed	274 (57.9%)	284 (58.2%)	558 (58.1%)	

Statistics for numeric variables are presented as mean (SD), while those for categorical variables are presented as count (%). Counts across levels of some categorical variables may not sum to the corresponding total due to missing values. N= number; P‐value, level of statistical significance.

### Correlations among liver biomarkers

**FIGURE 1:**
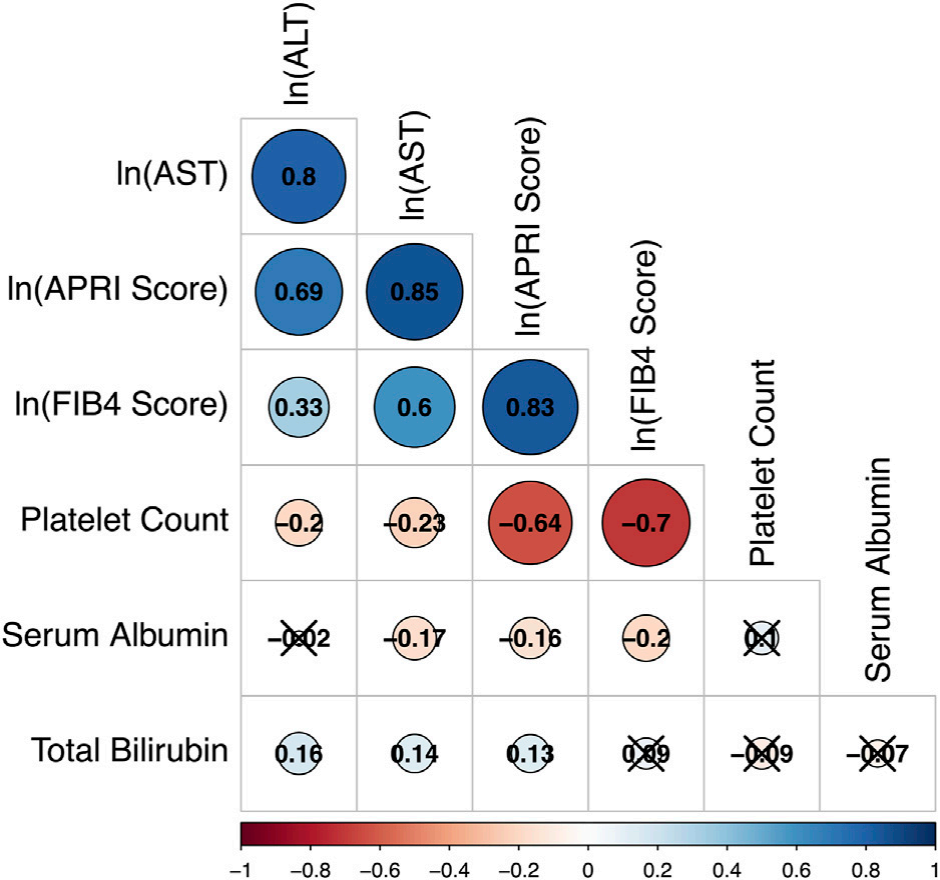
Correlation matrix based on Spearman coefficients for selected liver markers among all study participants. The strength and direction of each correlation is indicated by the size and color of its representative circle in the matrix. A legend for the colors is included beneath the matrix. Values of the Spearman correlation coefficients are also presented within the circles. Cross-off circles indicate that the observed correlation coefficient is not statistically significant after Bonferroni correction to an overall a = 0.05.

To illustrate the overall correlation between seven liver function markers, we summarized the pair-wise correlation in Figure 1. Observed values for ALT, AST, FIB-4 score, and APRI score all correlated positively and strongly with each other (Spearman correlation coefficient *ρ* > 0.6), except for ALT and FIB-4 score with moderate correlation (*ρ* = 0.36) (Figure 1). Platelet count correlated negatively with AST, ALT, FIB-4 score, and APRI score to varying degrees and weakly with albumin (*ρ* = 0.13), but not with total bilirubin (*ρ* = -0.06). Serum albumin correlated negatively with AST, APRI score, and FIB-4 score (−0.25 ≤ *ρ* ≤ −0.18) but not with ALT (*ρ* = −0.01). Total bilirubin did not correlate significantly with serum albumin, platelet count, FIB-4 score, or APRI score (*p* > 0.05) and correlated only weakly with AST and ALT (*ρ* < 0.14). Distributions of the biomarkers before and after transformation, are presented in Supplementary Figure S1.

### Covariate associations with liver biomarkers

Associations between covariates included in the final EWAS model and the selected liver biomarkers are presented in Table 2. Only “ever infection with HCV” was associated with all seven selected liver biomarkers when controlling for age, while diabetes status was not associated with any of the selected markers. All liver markers associated with at least four of the covariates included in the model excluding cell type proportions. Regardless of the *p*-value for the observed association between each covariate and each liver biomarker, all covariates were consistently included in the final epigenetic analysis model for each liver biomarker. Participants with complete data of all covariates were analyzed.

**TABLE 2:** Associations between DNA methylation age acceleration and selected biomarkers of liver function.

		Model			
Liver Marker		PhenoAA	GrimAA	IEAA	EEAA
ln (AST)	Beta (SE)	2.*5*×10^–3^ (2.1 × 10^–3^)	7.*9*×10^–3^ (4.0 × 10^–3^)	2.6 × 10^–3^ (2.8 × 10^–3^)	1.*9*×10^–3^ (2.8 × 10^–3^)
	p	0.23	0.05	0.36	0.51
ln (ALT)	Beta (SE)	1.9 × 10^–4^ (2.5 × 10^–3^)	3.4 × 10^–3^ (4.7 × 10^–3^)	1.3 × 10^–3^ (3.3 × 10^–3^)	5.5 × 10^–4^ (3.3 × 10^–3^)
	p	0.94	0.47	0.69	0.87
ALB	Beta (SE)	-7.0 × 10^–3^ (2.1 × 10^–3^)	-7.2 × 10^–3^ (4.0 × 10^–3^)	-4.1 × 10^–3^ (2.8 × 10^–3^)	-4.3 × 10^–3^ (2.8 × 10^–3^)
	p	8.6 × 10^–4^	0.07	0.15	0.12
TBILI	Beta (SE)	-4.9 × 10^–3^ (2.3 × 10^–3^)	-1.8 × 10^–3^ (4.4 × 10^–3^)	2.1 × 10^–3^ (3.2 × 10^–3^)	2.0 × 10^–3^ (3.1 × 10^–3^)
	p	0.04	0.69	0.50	0.52
PLT	Beta (SE)	-0.48 (0.32)	-0.12 (0.61)	-0.2 (0.44)	-0.72 (0.43)
	p	0.14	0.84	0.64	0.09
ln (FIB-4)	Beta (SE)	1.4 × 10^–3^ (2.2 × 10^–3^)	-5.5 × 10^–4^ (4.1 × 10^–3^)	-1.4 × 10^–3^ (2.9 × 10^–3^)	1.8 × 10^–3^ (2.9 × 10^–3^)
	p	0.53	0.89	0.63	0.54
ln (APRI)	Beta (SE)	1.3 × 10^–3^ (2.9 × 10^–3^)	3.2 × 10^–3^ (5.5 × 10^–3^)	2.6 × 10^–4^ (3.9 × 10^–3^)	3.7 × 10^–3^ (3.9 × 10^–3^)
	p	0.64	0.56	0.95	0.34

Biomarker values were ln-transformed when needed to achieve a more normal distribution as indicated on the left and then regressed on each DNA methylation age acceleration metric in linear models that control for the covariates included in the EWAS model. Associations that remained significant after FDR adjustment at Q < 0.05 are highlighted in yellow. Abbreviations: AST, aspartate aminotransferase (units/L); ALT, alanine aminotransferase (units/L); ALB, serum albumin (mg/dl); TBILI, total bilirubin level (mg/dl); PLT, platelet count (cells/µL); FIB-4, FIB-4 score; APRI, APRI score; PhenoAA, phenotype age acceleration; GrimaAA, Grim age acceleration; IEAA, intrinsic epigenetic age acceleration; EEAA, extrinsic epigenetic age acceleration.

### Associations of DNAm age acceleration with liver biomarkers

**FIGURE 2:**
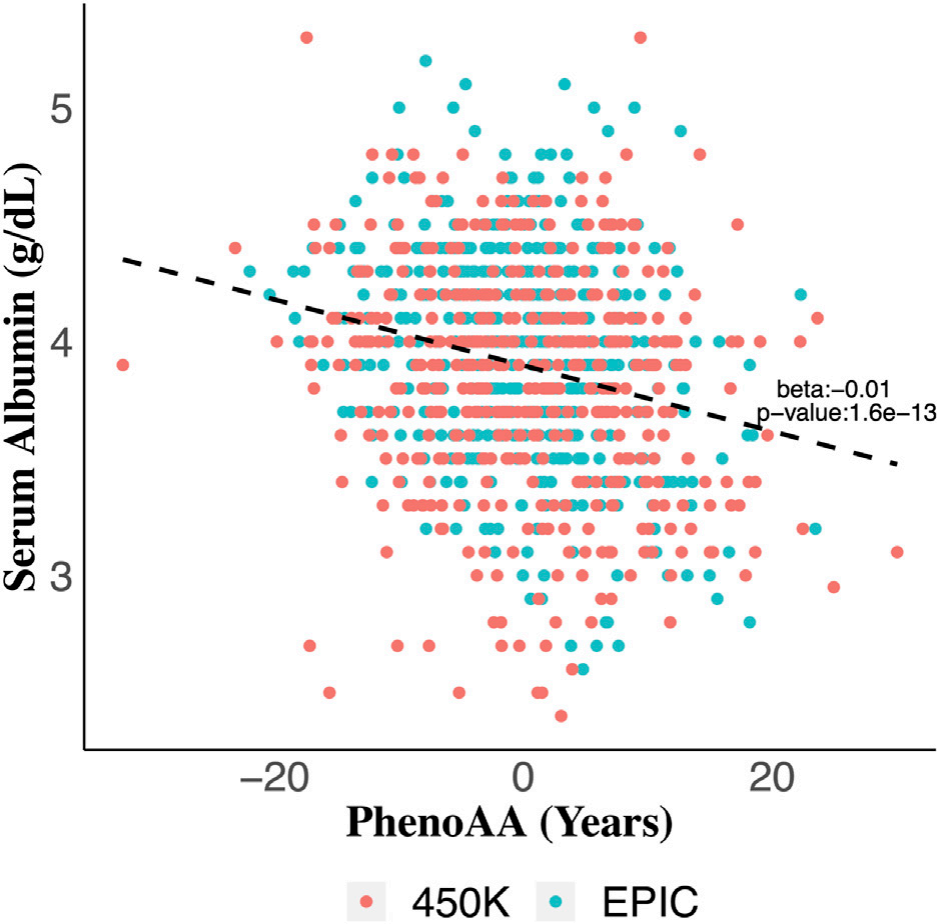
Scatter plot of serum albumin values across values for phenotypic age acceleration (PhenoAA) among the entire cohort. Red dots indicate participants from 450K and green dots indicate participants from EPIC. An unadjusted best-fit line is included (b = -0.01, *p*-value = 1.6 × 10^–13^).

Serum albumin was significantly associated with PhenoAA (beta = −0.014, *p*-value = 1.6 × 10^–13^) in an unadjusted model (Figure 2). That association remained significant after adjusting for all covariates in the final regression model (beta = −0.007, *p*-value = 8.6 × 10^–4^) (Table 3). No other metric of DNAm AA (i.e., IEAA, EEAA, and GrimAA) was associated with any other selected biomarker of liver function in the adjusted model (Table 3).

**TABLE 3:** DNAm sites are significantly associated with selected liver markers after meta-analysis and FDR correction.

Serum albumin										
DNAm	Chr	Pos	Gene	Meta-analysis			EPIC		450K	
				Beta (SE)	*p*	FDR	Beta (SE)	*p*	Beta (SE)	*p*
cg16936953	17	57915665	*TMEM49*	1.2 (0.21)	4.2 × 10^–9^	0.001	1.0 (0.29)	6.6 × 10^–4^	1.4 (0.29)	1.7 × 10^–6^
cg18181703	17	76354621	*SOCS3*	1.9 (0.35)	9.5 × 10^–8^	0.009	2.3 (0.45)	9.9 × 10^–7^	1.3 (0.55)	2.1 × 10^–2^
cg03546163	6	35654363	*FKBP5*	1.1 (0.20)	1.1 × 10^–7^	0.009	1.2 (0.26)	9.2 × 10^–6^	0.94 (0.33)	4.5 × 10^–3^
cg18942579	17	57915773	*TMEM49*	1.4 (0.26)	1.1 × 10^–7^	0.009	1.2 (0.34)	7.3 × 10^–4^	1.7 (0.41)	3.2 × 10^–5^
cg01409343	17	57915740	*TMEM49*	1.7 (0.32)	1.2 × 10^–7^	0.009	1.7 (0.43)	1.4 × 10^–4^	1.8 (0.48)	3.1 × 10^–4^
cg20995564	2	145172035	*ZEB2*	1.2 (0.23)	1.9 × 10^–7^	0.012	1.5 (0.29)	3.0 × 10^–7^	0.66 (0.39)	9.1 × 10^–2^
cg23966214	17	48203188	*SAMD14*	3.5 (0.70)	4.7 × 10^–7^	0.024	3.2 (0.81)	8.7 × 10^–5^	4.4 (1.4)	1.6 × 10^–3^
cg12054453	17	57915717	*TMEM49*	1.0 (0.20)	5.0 × 10^–7^	0.024	0.7 (0.29)	1.1 × 10^–2^	1.3 (0.28)	8.4 × 10^–6^
cg12992827	3	101901234	None Mapped	1.9 (0.38)	8.0 × 10^–7^	0.034	2.2 (0.47)	4.0 × 10^–6^	1.3 (0.66)	5.5 × 10^–2^

Chr: chromosome number; Pos: basepair position (human genome build hg19); beta: beta coefficient; SE: standard error; *p*: *p*-value, i.e. level of statistical significance. Results for DNAm sites where DNA methylation remained significantly associated with serum albumin after meta-analysis and FDR correction to Q < 0.05 are presented, including statistics from the meta-analysis and from the separate EWAS of the two cohorts. No significant associations were identified after meta-analysis and FDR correction for AST, ALT, total serum albumin, platelet count, FIB-4 score, or APRI score, so those markers are excluded from the table. coefficients represent the average change in serum albumin (mg/dl) expected for an increase in DNA methylation from 0% to 100%.

### EWAS and meta-analysis of liver biomarkers

**FIGURE 3:**
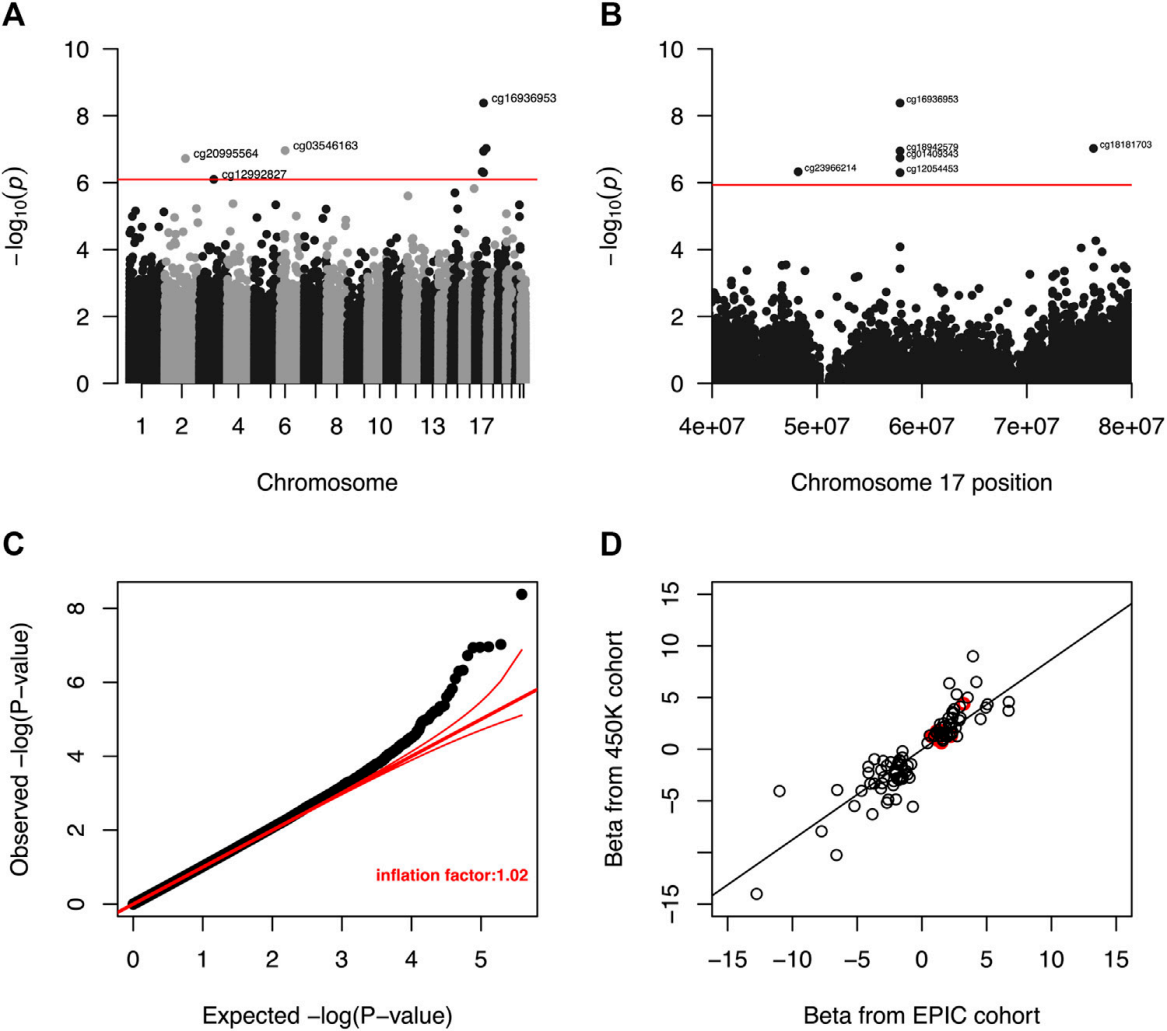
**(A)** Manhattan plot of unadjusted *p*-values from meta-analysis of EWAS results for serum albumin. The red line indicates the unadjusted *p*-value that corresponds to a threshold for FDR significance at Q < 0.05. DNA methylation was not significantly associated with serum albumin at any DNAm site after a more restrictive Bonferroni correction to an overall a = 0.05. **(B)** Regional Manhattan plot of unadjusted *p*-values from meta-analysis of EWAS results for CpG sites in chromosome 17 and serum albumin. The red line indicates the unadjusted *p*-value that corresponds to a threshold for FDR significance (among all CpG sites from across entire genome) at Q < 0.05. DNA methylation was not significantly associated with serum for any CpG site after a more restrictive Bonferroni correction to an overall a = 0.05. **(C)** Quantile-quantile plot of unadjusted *p*-values from meta-analysis of EWAS results for serum albumin. The global inflation factor was 1.02. Red lines represent a distribution of null associations with a 95% confidence interval. **(D)** Correlation of beta coefficients obtained from separate EWAS of serum albumin among the EPIC and 450K cohorts for the 100 DNAm sites with the smallest unadjusted *p*-values after meta-analysis. Beta coefficients from the separate analyses are positively correlated (r = 0.89, *p*-value < 0.001), indicating that the signs of observed beta coefficient are consistent between the two datasets. Circles representing *p*-value pairs that were significant after meta-analysis and FDR correction to Q < 0.05 are highlighted in red.

Meta-analysis of the EWAS results from the EPIC and 450K datasets for each selected liver biomarker and each DNAm site included in both platforms identified that hypermethylation at nine DNAm sites were significantly associated with increased serum albumin among male veterans with HIV after adjusting for all covariates (Table 3; Figure 3A). A regional plot for the section of chromosome 17 that contains five significant DNAm sites is presented in Figure 3B. Quantile-quantile analysis of the expected and observed *p*-values from the meta-analysis for serum albumin showed very moderate global inflation (inflation factor of 1.02) of the nominal *p*-values (Figure 3C), so no further corrections were applied. Notably, among the top one hundred DNAm sites most strongly associated with (i.e., with the lowest *p*-values) serum albumin in the meta-analysis, the beta coefficients obtained from the two EWAS sub-cohorts (the EPIC and 450K) show a strong, positive correlation (beta = 0.89, *p*-value < 0.001) (Figure 3D).

Four of the nine DNAm sites was positively associated with serum albumin—cg16936953, cg18942579, cg01409343, and cg12054453—annotated to be located within the *TMEM49* gene. A 10% increase in methylation at each of those sites corresponded to average increases in serum albumin of 0.12 g/dl (95% CI: 0.08, 0.16 g/dl), 0.14 g/dl (95% CI: 0.09, 0.19 g/dl), 0.17 g/dl (95% CI: 0.11, 0.23 g/dl), and 0.10 g/dl (95% CI: 0.06, 0.14 g/dl), respectively. Four of the remaining five DNAm sites—cg18181703, cg03546163, cg20995564, and cg23966214—annotated to correspond to the *SOCS3*, *FKBP5*, *ZEB2*, and *SAMD14* genes, respectively. A 10% increase in methylation at each of those DNAm sites corresponded to average increases in serum albumin of 0.19 g/dl (95% CI: 0.12, 0.26 g/dl), 0.11 g/dl (95% CI: 0.07, 0.15 g/dl), 0.12 g/dl (95% CI: 0.07, 0.17 g/dl), and 0.35 g/dl (95% CI: 0.21, 0.49 g/dl). The remaining DNAm site, cg12992827, was not annotated to correspond to any known coding sequence. A 10% increase in methylation at that site corresponded to an average increase in serum albumin of 0.19 g/dl (95% CI: 0.11, 0.26 g/dl). A quantile-quantile plot and Manhattan plot of the nominal *p-*values obtained from meta-analysis of the EPIC and 450K sub-cohorts are presented in Supplementary Figures S2, S3 for the other six liver biomarkers. Additionally, statistics and annotations for the ten DNAm sites with the lowest *p*-values after meta-analysis are presented in Supplementary Table S1 for each tested liver marker. Among the DNAm sites that were included in only one of the two methylation assay platforms used for this study, and that were not included in the meta-analysis, none was significantly associated with any of the liver biomarkers after correcting for multiple testing. Statistics and annotations for the ten DNAm sites with the lowest *p*-values among those that could not be meta-analyzed are presented for each liver biomarker in Supplementary Table S2.

## Discussion

In this study we evaluated the epigenetic associations with seven clinically relevant biomarkers of liver function—aspartate transaminase, alanine transaminase, serum albumin, total bilirubin, platelet count, FIB-4 score, and APRI score—among male United States veterans with HIV. We identified that nine DNAm sites mapped to the genes *TMEM49*, *SOCS3*, *FKBP5*, *ZEB2*, and *SAMD14* were significantly associated with serum albumin levels. We also demonstrate an association between higher PhenoAA (an aging marker for DNAm AA) and low serum levels of albumin.

Serum albumin is a highly abundant protein primarily synthesized by the liver. Synthesis of albumin occurs in polysomes of hepatocytes at a rate of 10 g/day–15 g/day and accounts of 10% of liver protein synthesis (Garcovich et al., 2009). Albumin is not stored in the liver and therefore is not released on demand. Instead in situations of increased need in healthy adults, hepatocytes can increase albumin synthesis by up to 300% (Garcovich et al., 2009). Impaired albumin synthesis and function have been reported in many liver diseases (Carvalho and Machado, 2018), and low serum levels of albumin may be a useful indicator of LD. Several studies have shown that serum albumin is an important prognostic factor and a significant predictor of death in patients with cirrhosis (Carvalho and Machado, 2018) and also a predictor of serious non-AIDS events in PWH (Ronit et al., 2018a; Ronit et al., 2018b).

Four of the nine serum albumin associated DNAm sites are located near the *TMEM49* gene (also known as *VMP1*). DNAm site *TMEM49*-cg16936953 was also associated with HIV infection, C-reactive protein and inflammatory bowel disease (Gross et al., 2016; Ligthart et al., 2016; Ventham et al., 2016). *TMEM49* encodes a transmembrane protein that drives cellular autophagy (Molejon et al., 2013) and is overexpressed in pancreatitis-affected acinar cells (Vaccaro et al., 2003). Cellular autophagy, therefore, may be linked to regulation of serum albumin. Previous studies have shown that serum albumin suppresses autophagy *via* mTOR activation and that depriving cultured cells of serum albumin induces autophagy which is protective against the accumulation of harmful reactive oxygen species (ROS).

*SOCS3* encodes a protein that inhibits cytokine signaling in the STAT pathway in response to elevated cytokine levels (Jo et al., 2005; Carow and Rottenberg, 2014). Suppression of *SOCS3* contributes to liver fibrosis by increasing fibrogenic signaling *via* STAT3-mediated upregulation of tissue growth factor (TGF)-β (Ogata et al., 2006; Jadid et al., 2018; Dees et al., 2020). In addition, obesity has been associated with downregulation of *SOCS3*, which raises the possibility that an association between DNAm near *SOCS3* and liver dysfunction could result from the well described relationship between obesity and liver dysfunction. DNAm site *SOCS3*-cg18181703 was associated with BMI in several recent EWAS (Ali et al., 2016; Mendelson et al., 2017; Wahl et al., 2017; Xu et al., 2018; Sun et al., 2019). In this cohort of PWH, *SOCS3*-cg18181703 was associated with BMI (*p*-value:0.044) further supporting this hypothesis. Epigenetic associations between *SOCS3*-cg18181703 and C-reactive protein, inflammatory bowel disease, type-2 diabetes and cognitive abilities (Chambers et al., 2015; Ligthart et al., 2016; Marioni et al., 2018; Juvinao-Quintero et al., 2021) also suggest potential pleiotropic effects of *SOCS3* gene.

*FKBP5* encodes an immunoregulatory protein that contributes to basic protein folding and trafficking (Zannas et al., 2016). *FKBP5* has been demonstrated to positively regulate the stress response and drive acquisition of metabolic disorders including obesity, insulin resistance, and diabetes (Sidibeh et al., 2018). Moreover, *FKBP5* is thought to contribute to liver dysfunction (Kusumanchi et al., 2021) and in one study, deletion of *FKBP5* protected knock-out mice from fatty liver disease despite high fat diets (Stechschulte et al., 2016). *ZEB2* encodes a zinc-finger DNA-binding protein expressed in hepatocytes (Cai et al., 2012). Studies have demonstrated that repression of *ZEB2* expression by microRNAs induces apoptosis in hepatocytes (Zhao et al., 2018), suggesting that *ZEB2* might influence preservation of liver integrity in the face of hepatocellular damage. The remaining DNAm site associated positively with serum albumin levels was located near the *SMAD14* gene. The function of the *SMAD14* gene is unknown, but it has been linked to gastric cancer and hypothesized to act as a tumor suppressor gene (Xu et al., 2020). No studies have associated *SMAD14* or its tumor suppressor properties to either general liver health and function or specifically to serum albumin regulation.

Despite the association between PhenoAA and serum albumin, no other significant associations were noted between the four assessed metrics of DNAm AA—PhenoAA, GrimAA, IEAA, and EEAA—and other biomarkers. Compared to the other metrics of DNAm aging, PhenoAA is unique in that it is highly predictive of morbidity and mortality even after adjusting for chronological age (Liu et al., 2019). This may explain its significant association with albumin, which is also a prognostic marker of survival in LD (Carvalho and Verdelho Machado, 2018). It is plausible that specific DNAm sites may regulate the expression of genes in a manner that influences behavior of the selected biomarkers of liver health, either by impacting the progression of conditions that damage the liver and inhibit function—like fibrosis or hepatocellular carcinoma—or by affecting pathways that are specifically involved in regulation of a specific liver biomarkers.

Low serum level of albumin, though an important prognostic biomarker for LD (Carvalho and Verdelho Machado, 2018), is not specific. Thus, the associations we have uncovered in this study may be mediated by other non-hepatic factors in PWH. For example, hypoalbuminemia is associated with inflammation and synthesis of albumin is suppressed by pro-inflammatory cytokines TNF-α and Interleukin-1 (Dinarello, 1984; Perlmutter et al., 1986), both of which are important mediators of chronic inflammation in PWH (Deeks et al., 2013; De Pablo-Bernal et al., 2014). It is thus presumptuous to infer that the association between DNAm and albumin reflects underlying LD alone and is not combination of multiple interacting factors.

### Strengths and limitations

This study combines data from two different microarray platforms to assess consistent epigenetic associations of overlapping DNAm sites. This enabled a robust meta-analysis of epigenetic associations with a large sample size. The significant associations are likely to be true positive findings, and should be replicated in future studies. Secondly, we examine multiple biomarkers which when combined reflect overall liver function and individually inform on the liver’s capacity to perform specific metabolic functions. This allowed us to examine DNAm sites across a wider range of genes that may link to specific aspects of liver function. Thirdly, unlike other EWAS of liver function or LD, our cohort included exclusively people with HIV, a group which has a higher burden of liver dysfunction and related morbidity. Our findings contribute to the identification of genes that may be relevant to liver health and function in the context of HIV infection.

One limitation of our study is that EWAS discovery was sub-optimally powered for a subset of DNAm sites included in only one of the platforms. Meta-analysis results from DNAm sites measured on both platforms were highly consistent, suggesting that combining data from the two platforms successfully augments the power to detect significant associations. Future replication and meta-analysis with extended epigenomic coverage would improve the discovery of liver function-associated DNAm sites. Secondly, our study population was restricted to male veterans with HIV, limiting the generalizability of our findings to women with HIV. Thirdly, we used DNAm levels measured in blood cells to assess associations between DNAm and biomarkers of liver function, which may not represent DNAm patterns in the liver cells that are responsible for regulating these biomarkers. The lack of significant associations between DNAm and AST, ALT, total serum bilirubin, platelet count, FIB-4 score and, APRI score might result from an absence of epigenetic associations with liver functions of large effects, suggesting better powered future studies are needed to identify epigenetic associations with liver functions with smaller effects. Also, the potential for misclassifying liver fibrosis with the use of clinical scores like FIB-4 and APRI as has been shown in hepatitis B infection (Kim et al., 2016b) and may have limited our ability to detect an association with these markers.

## Conclusion

We identified epigenetic associations of both individual DNAm sites and DNAm AA with liver function through serum albumin in men with HIV. EWAS may inform on disease pathogenesis and generate new hypotheses for predicting disease progression and treatment of LD in HIV. Identifying specific genes linked with liver function among PWH can inform disease progression to improve the health of this patient population. Further replication analyses in independent cohorts are warranted to confirm and improve the discovery of the epigenetic markers of liver function and LD in PWH.

## Data Availability

The data analyzed in this study is subject to the following licenses/restrictions: Due to US Department of Veterans Affairs (VA) regulations and our ethics agreements, the analytic data sets used for this study are not permitted to leave the VA firewall without a Data Use Agreement. This limitation is consistent with other studies based on VA data. However, VA data are made freely available to researchers with an approved VA study protocol. For more information, please visit https://www.virec.research.va.gov or contact the VA Information Resource Center at VIReC@va.gov. Requests to access these datasets should be directed to yan.v.sun@emory.edu.

## Abbreviations

AA, Age acceleration; ALT, Alanine amino transferase; APRI, AST to platelet ratio index; ART, Antiretroviral therapy; AST, Aspartate amino transferase; BMI, Body mass index; CpG, 5′—Cytosine—phosphate—Guanine—3′; DNAm, Deoxyribonucleic acid methylation; EEAA, Extrinsic epigenetic age acceleration; EWAS, Epigenome-wide association study; FIB, Fibrosis; FKBP, FK506 binding protein; HBV, Hepatitis B virus; HCC, Hepatocellular carcinoma; HCV, Hepatitis C virus; HIV, Human immunodeficiency virus; IEAA, Intrinsic epigenetic age acceleration; LD, Liver Disease; NACD, Non-AIDS related chronic disease; NAFLD, Non-Alcohol fatty liver disease; PWH, People with HIV; SOCS, suppressor of cytokine signaling; STAT, Signal transducer and activator of transcription; TGF, Tissue growth factor; TMEM, transmembrane; TNFα, tumor necrosis factor- alpha; VACS, Veterans aging cohort; ZEB, Zinc Finger E-Box Binding Homeobox 1.

## References

[B1] Ali O. Cerjak D. Kent J. W. Jr. James R. Blangero J. Carless M. A. (2016). Methylation of SOCS3 is inversely associated with metabolic syndrome in an epigenome-wide association study of obesity. Epigenetics 11, 699–707. 10.1080/15592294.2016.1216284 27564309 PMC5048720

[B2] Bell C. G. Lowe R. Adams P. D. Baccarelli A. A. Beck S. Bell J. T. (2019). DNA methylation aging clocks: Challenges and recommendations. Genome Biol. 20, 249. 10.1186/s13059-019-1824-y 31767039 PMC6876109

[B3] Cai M. Y. Luo R. Z. Chen J. W. Pei X. Q. Lu J. B. Hou J. H. (2012). Overexpression of ZEB2 in peritumoral liver tissue correlates with favorable survival after curative resection of hepatocellular carcinoma. PLoS One 7, e32838. 10.1371/journal.pone.0032838 22393452 PMC3290607

[B4] Carow B. Rottenberg M. E. (2014). SOCS3, a major regulator of infection and inflammation. Front. Immunol. 5, 58. 10.3389/fimmu.2014.00058 24600449 PMC3928676

[B5] Carvalho J. R. Machado M. V. (2018). New insights about albumin and liver disease. Ann. Hepatol. 17, 547–560. 10.5604/01.3001.0012.0916 29893696

[B6] Carvalho J. R. Verdelho Machado M. (2018). New insights about albumin and liver disease. Ann. Hepatol. 17, 547–560. 10.5604/01.3001.0012.0916 29893696

[B7] Chambers J. C. Loh M. Lehne B. Drong A. Kriebel J. Motta V. (2015). Epigenome-wide association of DNA methylation markers in peripheral blood from Indian asians and Europeans with incident type 2 diabetes: A nested case-control study. Lancet. Diabetes Endocrinol. 3, 526–534. 10.1016/S2213-8587(15)00127-8 26095709 PMC4724884

[B8] Chen B. H. Marioni R. E. Colicino E. Peters M. J. Ward-Caviness C. K. Tsai P. C. (2016). DNA methylation-based measures of biological age: meta-analysis predicting time to death. Aging (Albany NY) 8, 1844–1865. 10.18632/aging.101020 27690265 PMC5076441

[B9] Chen J. Huang Y. Hui Q. Mathur R. Gwinn M. So-Armah K. (2020). Epigenetic associations with estimated glomerular filtration rate among men with human immunodeficiency virus infection. Clin. Infect. Dis. 70, 667–673. 10.1093/cid/ciz240 30893429 PMC7319269

[B10] De Pablo-Bernal R. S. Ruiz-Mateos E. Rosado I. Dominguez-Molina B. Alvarez-Rios A. I. Carrillo-Vico A. (2014). TNF-α levels in HIV-infected patients after long-term suppressive cART persist as high as in elderly, HIV-uninfected subjects. J. Antimicrob. Chemother. 69, 3041–3046. 10.1093/jac/dku263 25011654

[B11] Deeks S. G. Tracy R. Douek D. C. (2013). Systemic effects of inflammation on health during chronic HIV infection. Immunity 39, 633–645. 10.1016/j.immuni.2013.10.001 24138880 PMC4012895

[B12] Dees C. Pötter S. Zhang Y. Bergmann C. Zhou X. Luber M. (2020). TGF-β-induced epigenetic deregulation of SOCS3 facilitates STAT3 signaling to promote fibrosis. J. Clin. Invest. 130, 2347–2363. 10.1172/JCI122462 31990678 PMC7190914

[B13] Dinarello C. A. (1984). Interleukin-1 and the pathogenesis of the acute-phase response. N. Engl. J. Med. 311, 1413–1418. 10.1056/NEJM198411293112205 6208485

[B14] Freiberg M. S. McGinnis K. A. Kraemer K. Samet J. H. Conigliaro J. CuRtis Ellison R. (1999). The association between alcohol consumption and prevalent cardiovascular diseases among HIV-infected and HIV-uninfected men. J. Acquir. Immune Defic. Syndr. 53, 247–253. 10.1097/QAI.0b013e3181c6c4b7 PMC2858978 20009766

[B15] French S. W. (2013). Epigenetic events in liver cancer resulting from alcoholic liver disease. Alcohol Res. 35, 57–67. 24313165 10.35946/arcr.v35.1.07 PMC3860418

[B16] Garcovich M. Zocco M. A. Gasbarrini A. (2009). Clinical use of albumin in hepatology. Blood Transfus. 7, 268–277. 10.2450/2008.0080-08 20011638 PMC2782804

[B17] Gross A. M. Jaeger P. A. Kreisberg J. F. Licon K. Jepsen K. L. Khosroheidari M. (2016). Methylome-wide analysis of chronic HIV infection reveals five-year increase in biological age and epigenetic targeting of HLA. Mol. Cell 62, 157–168. 10.1016/j.molcel.2016.03.019 27105112 PMC4995115

[B18] Hannum G. Guinney J. Zhao L. Zhang L. Hughes G. Sadda S. (2013). Genome-wide methylation profiles reveal quantitative views of human aging rates. Mol. Cell 49, 359–367. 10.1016/j.molcel.2012.10.016 23177740 PMC3780611

[B19] Horvath S. Levine A. J. (2015). HIV-1 infection accelerates age according to the epigenetic clock. J. Infect. Dis. 212, 1563–1573. 10.1093/infdis/jiv277 25969563 PMC4621253

[B20] Houseman E. A. Accomando W. P. Koestler D. C. Christensen B. C. Marsit C. J. Nelson H. H. (2012). DNA methylation arrays as surrogate measures of cell mixture distribution. BMC Bioinforma. 13, 86. 10.1186/1471-2105-13-86 PMC3532182 22568884

[B21] Jadid F. Z. Chihab H. Alj H. S. Elfihry R. Zaidane I. Tazi S. (2018). Control of progression towards liver fibrosis and hepatocellular carcinoma by SOCS3 polymorphisms in chronic HCV-infected patients. Infect. Genet. Evol. 66, 1–8. 10.1016/j.meegid.2018.08.027 30172885

[B22] Jo D. Liu D. Yao S. Collins R. D. Hawiger J. (2005). Intracellular protein therapy with SOCS3 inhibits inflammation and apoptosis. Nat. Med. 11, 892–898. 10.1038/nm1269 16007096

[B23] Justice A. C. Dombrowski E. Conigliaro J. Fultz S. L. Gibson D. Madenwald T. (2006). Veterans aging cohort study (VACS): Overview and description. Med. Care 44, S13–S24. 10.1097/01.mlr.0000223741.02074.66 16849964 PMC3049942

[B24] Juvinao-Quintero D. L. Marioni R. E. Ochoa-Rosales C. Russ T. C. Deary I. J. van Meurs J. B. J. (2021). DNA methylation of blood cells is associated with prevalent type 2 diabetes in a meta-analysis of four European cohorts. Clin. Epigenetics 13, 40. 10.1186/s13148-021-01027-3 33622391 PMC7903628

[B25] Kaspar M. B. Sterling R. K. (2017). Mechanisms of liver disease in patients infected with HIV. BMJ Open Gastroenterol. 4, e000166. 10.1136/bmjgast-2017-000166 PMC5663263 29119002

[B26] Kim H. N. Nance R. Van Rompaey S. Delaney J. C. Crane H. M. Cachay E. R. (2016). Poorly controlled HIV infection: An independent risk factor for liver fibrosis. J. Acquir. Immune Defic. Syndr. 72, 437–443. 10.1097/QAI.0000000000000992 26990826 PMC4925189

[B27] Kim W. R. Berg T. Asselah T. Flisiak R. Fung S. Gordon S. C. (2016). Evaluation of APRI and FIB-4 scoring systems for non-invasive assessment of hepatic fibrosis in chronic Hepatitis B patients. J. Hepatol. 64, 773–780. 10.1016/j.jhep.2015.11.012 26626497

[B28] Konopnicki D. Mocroft A. de Wit S. Antunes F. Ledergerber B. Katlama C. (2005). Hepatitis B and HIV: Prevalence, AIDS progression, response to highly active antiretroviral therapy and increased mortality in the EuroSIDA cohort. AIDS 19, 593–601. 10.1097/01.aids.0000163936.99401.fe 15802978

[B29] Kusumanchi P. Liang T. Zhang T. Ross R. A. Han S. Chandler K. (2021). Stress-responsive gene FK506-binding protein 51 mediates alcohol-induced liver injury through the hippo pathway and chemokine (C-X-C motif) ligand 1 signaling. Hepatology 74, 1234–1250. 10.1002/hep.31800 33710653 PMC8435051

[B30] Lemoine M. Serfaty L. Capeau J. (2012). From nonalcoholic fatty liver to nonalcoholic steatohepatitis and cirrhosis in HIV-infected patients: Diagnosis and management. Curr. Opin. Infect. Dis. 25, 10–16. 10.1097/QCO.0b013e32834ef599 22183113

[B31] Levine M. E. Hosgood H. D. Chen B. Absher D. Assimes T. Horvath S. (2015). DNA methylation age of blood predicts future onset of lung cancer in the women's health initiative. Aging (Albany NY) 7, 690–700. 10.18632/aging.100809 26411804 PMC4600626

[B32] Levine M. E. Lu A. T. Bennett D. A. Horvath S. (2015). Epigenetic age of the pre-frontal cortex is associated with neuritic plaques, amyloid load, and Alzheimer's disease related cognitive functioning. Aging (Albany NY) 7, 1198–1211. 10.18632/aging.100864 26684672 PMC4712342

[B33] Levine M. E. Lu A. T. Quach A. Chen B. H. Assimes T. L. Bandinelli S. (2018). An epigenetic biomarker of aging for lifespan and healthspan. Aging (Albany NY) 10, 573–591. 10.18632/aging.101414 29676998 PMC5940111

[B34] Ligthart S. Marzi C. Aslibekyan S. Mendelson M. M. Conneely K. N. Tanaka T. (2016). DNA methylation signatures of chronic low-grade inflammation are associated with complex diseases. Genome Biol. 17, 255. 10.1186/s13059-016-1119-5 27955697 PMC5151130

[B35] Liu Z. Kuo P-L. Horvath S. Crimmins E. Ferrucci L. Levine M. (2019). A new aging measure captures morbidity and mortality risk across diverse subpopulations from nhanes IV: A cohort study. PLoS Med. 15, e1002718. 10.1371/journal.pmed.1002718 PMC6312200 30596641

[B36] Lu A. T. Quach A. Wilson J. G. Reiner A. P. Aviv A. Raj K. (2019). DNA methylation GrimAge strongly predicts lifespan and healthspan. Aging (Albany NY) 11, 303–327. 10.18632/aging.101684 30669119 PMC6366976

[B37] Marioni R. E. McRae A. F. Bressler J. Colicino E. Hannon E. Li S. (2018). Meta-analysis of epigenome-wide association studies of cognitive abilities. Mol. Psychiatry 23, 2133–2144. 10.1038/s41380-017-0008-y 29311653 PMC6035894

[B38] Mathur R. Hui Q. Huang Y. Gwinn M. So-Armah K. Freiberg M. S. (2019). DNA methylation markers of type 2 diabetes mellitus among male veterans with or without human immunodeficiency virus infection. J. Infect. Dis. 219, 1959–1962. 10.1093/infdis/jiz023 30649532 PMC6534194

[B39] Mendelson M. M. Marioni R. E. Joehanes R. Liu C. Hedman A. K. Aslibekyan S. (2017). Association of body mass index with DNA methylation and gene expression in blood cells and relations to cardiometabolic disease: A mendelian randomization approach. PLoS Med. 14, e1002215. 10.1371/journal.pmed.1002215 28095459 PMC5240936

[B40] Molejon M. I. Ropolo A. Re A. L. Boggio V. Vaccaro M. I. (2013). The VMP1-Beclin 1 interaction regulates autophagy induction. Sci. Rep. 3, 1055. 10.1038/srep01055 23316280 PMC3542764

[B41] Moran S. Arribas C. Esteller M. (2016). Validation of a DNA methylation microarray for 850, 000 CpG sites of the human genome enriched in enhancer sequences. Epigenomics 8, 389–399. 10.2217/epi.15.114 26673039 PMC4864062

[B42] Nishida N. Nagasaka T. Nishimura T. Ikai I. Boland C. R. Goel A. (2008). Aberrant methylation of multiple tumor suppressor genes in aging liver, chronic hepatitis, and hepatocellular carcinoma. Hepatology 47, 908–918. 10.1002/hep.22110 18161048 PMC2865182

[B43] Ogata H. Chinen T. Yoshida T. Kinjyo I. Takaesu G. ShiraisHi H. (2006). Loss of SOCS3 in the liver promotes fibrosis by enhancing STAT3-mediated TGF-beta1 production. Oncogene 25, 2520–2530. 10.1038/sj.onc.1209281 16474852

[B44] Palella F. J. Baker R. K. Moorman A. C. Chmiel J. S. Wood K. C. Brooks J. T. (2006). Mortality in the highly active antiretroviral therapy era: Changing causes of death and disease in the HIV outpatient study. J. Acquir. Immune Defic. Syndr. 43, 27–34. 10.1097/01.qai.0000233310.90484.16 16878047

[B45] Perlmutter D. H. Dinarello C. A. Punsal P. I. Colten H. R. (1986). Cachectin/tumor necrosis factor regulates hepatic acute-phase gene expression. J. Clin. Invest. 78, 1349–1354. 10.1172/JCI112721 2429991 PMC423831

[B46] Rickabaugh T. M. Baxter R. M. Sehl M. Sinsheimer J. S. Hultin P. M. Hultin L. E. (2015). Acceleration of age-associated methylation patterns in HIV-1-infected adults. PLoS One 10, e0119201. 10.1371/journal.pone.0119201 25807146 PMC4373843

[B47] Ronit A. Hatleberg C. I. Ryom L. Bonnet F. El-Sadr W. Reiss P. (2018). Associations between serum albumin and serious non-AIDS events among people living with HIV. AIDS 32, 1837–1848. 10.1097/QAD.0000000000001900 29847331

[B48] Ronit A. Sharma S. Baker J. V. Mngqibisa R. Delory T. Caldeira L. (2018). Serum albumin as a prognostic marker for serious non-AIDS endpoints in the strategic timing of antiretroviral treatment (START) study. J. Infect. Dis. 217, 405–412. 10.1093/infdis/jix350 29244111 PMC5853310

[B49] Rosen A. D. Robertson K. D. Hlady R. A. Muench C. Lee J. Philibert R. (2018). DNA methylation age is accelerated in alcohol dependence. Transl. Psychiatry 8, 182. 10.1038/s41398-018-0233-4 30185790 PMC6125381

[B50] Shepard C. W. Finelli L. Alter M. J. (2005). Global epidemiology of hepatitis C virus infection. Lancet. Infect. Dis. 5, 558–567. 10.1016/S1473-3099(05)70216-4 16122679

[B51] Sidibeh C. O. Pereira M. J. Abalo X. M. J Boersma G. Skrtic S. Lundkvist P. (2018). FKBP5 expression in human adipose tissue: Potential role in glucose and lipid metabolism, adipogenesis and type 2 diabetes. Endocrine 62, 116–128. 10.1007/s12020-018-1674-5 30032404 PMC6153563

[B52] Smith C. Sabin C. A. Lundgren J. D. Thiebaut R. Weber R. (2010). Factors associated with specific causes of death amongst HIV-positive individuals in the D:A:D Study. AIDS 24, 1537–1548. 10.1097/QAD.0b013e32833a0918 20453631

[B53] Smith C. J. Ryom L. Weber R. Morlat P. Pradier C. Reiss P. (2014). Trends in underlying causes of death in people with HIV from 1999 to 2011 (D:A:D): A multicohort collaboration. Lancet 384, 241–248. 10.1016/S0140-6736(14)60604-8 25042234

[B54] Solomon O. MacIsaac J. Quach H. Tindula G. Kobor M. S. Huen K. (2018). Comparison of DNA methylation measured by Illumina 450K and EPIC BeadChips in blood of newborns and 14-year-old children. Epigenetics 13, 655–664. 10.1080/15592294.2018.1497386 30044683 PMC6140901

[B55] Stechschulte L. A. Qiu B. Warrier M. Hinds T. D. Zhang M. Gu H. (2016). FKBP51 null mice are resistant to diet-induced obesity and the PPARγ agonist rosiglitazone. Endocrinology 157, 3888–3900. 10.1210/en.2015-1996 27442117 PMC5045506

[B56] Sterling R. K. Lissen E. Clumeck N. Sola R. Correa M. C. Montaner J. (2006). Development of a simple noninvasive index to predict significant fibrosis in patients with HIV/HCV coinfection. Hepatology 43, 1317–1325. 10.1002/hep.21178 16729309

[B57] Su P. F. Lee T. C. Lin P. J. Lee P. H. Jeng Y. M. Chen C. H. (2007). Differential DNA methylation associated with Hepatitis B virus infection in hepatocellular carcinoma. Int. J. Cancer 121, 1257–1264. 10.1002/ijc.22849 17534893

[B58] Sun D. Zhang T. Su S. Hao G. Chen T. Li Q. Z. (2019). Body mass index drives changes in DNA methylation: A longitudinal study. Circ. Res. 125, 824–833. 10.1161/CIRCRESAHA.119.315397 31510868 PMC6786955

[B59] Vaccaro M. I. Grasso D. Ropolo A. Iovanna J. L. Cerquetti M. C. (2003). VMP1 expression correlates with acinar cell cytoplasmic vacuolization in arginine-induced acute pancreatitis. Pancreatology 3, 69–74. 10.1159/000069150 12649568

[B60] Varela-Rey M. Woodhoo A. Martinez-Chantar M. L. Mato J. M. Lu S. C. (2013). Alcohol, DNA methylation, and cancer. Alcohol Res. 35, 25–35. 24313162 10.35946/arcr.v35.1.04 PMC3860423

[B61] Ventham N. T. Kennedy N. A. Adams A. T. Kalla R. Heath S. O'Leary K. R. (2016). Integrative epigenome-wide analysis demonstrates that DNA methylation may mediate genetic risk in inflammatory bowel disease. Nat. Commun. 7, 13507. 10.1038/ncomms13507 27886173 PMC5133631

[B62] Wahl S. Drong A. Lehne B. Loh M. Scott W. R. Kunze S. (2017). Epigenome-wide association study of body mass index, and the adverse outcomes of adiposity. Nature 541, 81–86. 10.1038/nature20784 28002404 PMC5570525

[B63] Wai C. T. Greenson J. K. Fontana R. J. Kalbfleisch J. D. Marrero J. A. Conjeevaram H. S. (2003). A simple noninvasive index can predict both significant fibrosis and cirrhosis in patients with chronic hepatitis C. Hepatology 38, 518–526. 10.1053/jhep.2003.50346 12883497

[B64] Xu K. Zhang X. Wang Z. Hu Y. Sinha R. (2018). Epigenome-wide association analysis revealed that SOCS3 methylation influences the effect of cumulative stress on obesity. Biol. Psychol. 131, 63–71. 10.1016/j.biopsycho.2016.11.001 27826092 PMC5419875

[B65] Xu X. Chang X. Xu Y. Deng P. Wang J. Zhang C. (2020). SAMD14 promoter methylation is strongly associated with gene expression and poor prognosis in gastric cancer. Int. J. Clin. Oncol. 25, 1105–1114. 10.1007/s10147-020-01647-4 32206938

[B66] Zakhari S. (2013). Alcohol metabolism and epigenetics changes. Alcohol Res. 35, 6–16. 24313160 10.35946/arcr.v35.1.02 PMC3860421

[B67] Zannas A. S. Wiechmann T. Gassen N. C. Binder E. B. (2016). Gene-stress-epigenetic regulation of FKBP5: Clinical and translational implications. Neuropsychopharmacology 41, 261–274. 10.1038/npp.2015.235 26250598 PMC4677131

[B68] Zhang X. Asllanaj E. Amiri M. Portilla-Fernandez E. Bramer W. M. Nano J. (2021). Deciphering the role of epigenetic modifications in fatty liver disease: A systematic review. Eur. J. Clin. Invest. 51, e13479. 10.1111/eci.13479 33350463 PMC8243926

[B69] Zhang X. Hu Y. Justice A. C. Li B. Wang Z. Zhao H. (2017). DNA methylation signatures of illicit drug injection and hepatitis C are associated with HIV frailty. Nat. Commun. 8, 2243. 10.1038/s41467-017-02326-1 29269866 PMC5740109

[B70] Zhao Y. Xue F. Sun J. Guo S. Zhang H. Qiu B. (2014). Genome-wide methylation profiling of the different stages of Hepatitis B virus-related hepatocellular carcinoma development in plasma cell-free DNA reveals potential biomarkers for early detection and high-risk monitoring of hepatocellular carcinoma. Clin. Epigenetics 6, 30. 10.1186/1868-7083-6-30 25859288 PMC4391300

[B71] Zhao Y. X. Sun Y. Y. Huang A. L. Li X. F. Huang C. Ma T. T. (2018). MicroRNA-200a induces apoptosis by targeting ZEB2 in alcoholic liver disease. Cell Cycle 17, 250–262. 10.1080/15384101.2017.1417708 29251244 PMC5884358

