# Supplementary material for: Epigenome-wide association study of biomarkers of liver function identifies albumin-associated DNA methylation sites among male veterans with HIV
Source: Front Genet. 2022 Oct 11;13:1020871. doi: 10.3389/fgene.2022.1020871 (PMC9592923; doi:10.3389/fgene.2022.1020871)
Supplement: Supplementary file 2 [file Table1.docx]

**Supplementary Figure 1**:

Distributions of values for selected biomarkers of liver function among the entire cohort. Only biomarkers with highly right-skewed distributions were log-transformed to produce more normal distributions that allow for inference from linear modelling. For this figure, log2 transformation was used for easy interpretation. For EWAS, a natural log transformation was applied when needed. A) Distribution of AST before and after log transformation. B) Distribution of ALT before and after log transformation. C) Distributions of ALB without log transformation. D) Distributions of ALB without log transformation. E) Distributions of ALB without log transformation. F) Distribution of FIB-4 scores before and after log transformation. G) Distribution of APRI scores before and after log transformation. Abbreviations: AST, aspartate aminotransferase level (units/L); ALT, alanine aminotransferase level (units/L); ALB, serum albumin level (mg/dL); TBILI, total bilirubin level (mg/dL); PLT, platelet count (cells/mL); FIB-4, FIB-4 score; APRI, APRI score.

**Supplementary Figure 2**:

Regional Manhattan plot of unadjusted P-values from meta-analysis of EWAS results for CpG sites in chromosome 17 and serum albumin. The red line indicates the unadjusted P-value that corresponds to a threshold for FDR significance (among all CpG sites from across entire genome) at Q < 0.05. DNA methylation was not significantly associated with serum for any CpG site after a more restrictive Bonferroni correction to an overall a = 0.05.

**Supplementary Figure 3**:

Distribution of unadjusted P-values from meta-analysis of EWAS results from the EPIC and 450K cohorts for AST. (A) Quantile-quantile plot of unadjusted P-values from meta-analysis of EWAS results for AST. The global inflation factor was 1.04, so no further adjustment for global inflation was performed. Red lines represent a perfect 1:1 association with a 95% confidence interval. (B) Manhattan plot of unadjusted P-values from meta-analysis of EWAS results for AST. The red line indicates the unadjusted P-value that corresponds to a threshold for FDR significance at Q < 0.05. DNA methylation was not significantly associated with AST for any CpG site after a more restrictive Bonferroni correction to an overall a = 0.05.

**Supplementary Figure 4**:

Distribution of unadjusted P-values from meta-analysis of EWAS results from the EPIC and 450K cohorts for ALT. (A) Quantile-quantile plot of unadjusted P-values from meta-analysis of EWAS results for ALT. The global inflation factor was 1.14, so no further adjustment for global inflation was performed. Red lines represent a perfect 1:1 association with a 95% confidence interval. (B) Manhattan plot of unadjusted P-values from meta-analysis of EWAS results for ALT. The red line indicates the unadjusted P-value that corresponds to a threshold for FDR significance at Q < 0.05. DNA methylation was not significantly associated with ALT for any CpG site after a more restrictive Bonferroni correction to an overall a = 0.05.

**Supplementary Figure 5**: Distribution of unadjusted P-values from meta-analysis of EWAS results from the EPIC and 450K cohorts for total bilirubin. (A) Quantile-quantile plot of unadjusted P-values from meta-analysis of EWAS results for total bilirubin. The global inflation factor was 1.04, so no further adjustment for global inflation was performed. Red lines represent a perfect 1:1 association with a 95% confidence interval. (B) Manhattan plot of unadjusted P-values from meta-analysis of EWAS results for total bilirubin. The red line indicates the unadjusted P-value that corresponds to a threshold for FDR significance at Q < 0.05. DNA methylation was not significantly associated with total bilirubin for any CpG site after a more restrictive Bonferroni correction to an overall a = 0.05.

**Supplementary Figure 6**:

Distribution of unadjusted P-values from meta-analysis of EWAS results from the EPIC and 450K cohorts for platelet count. (A) Quantile-quantile plot of unadjusted P-values from meta-analysis of EWAS results for platelet count. The global inflation factor was 1.02, so no further adjustment for global inflation was performed. Red lines represent a perfect 1:1 association with a 95% confidence interval. (B) Manhattan plot of unadjusted P-values from meta-analysis of EWAS results for platelet count. The red line indicates the unadjusted P-value that corresponds to a threshold for FDR significance at Q < 0.05. DNA methylation was not significantly associated with platelet count for any CpG site after a more restrictive Bonferroni correction to an overall a = 0.05.

**Supplementary Figure 7**:

Distribution of unadjusted P-values from meta-analysis of EWAS results from the EPIC and 450K cohorts for FIB-4 score. (A) Quantile-quantile plot of unadjusted P-values from meta-analysis of EWAS results for FIB-4 score. The global inflation factor was 0.96, so no further adjustment for global inflation was performed. Red lines represent a perfect 1:1 association with a 95% confidence interval. (B) Manhattan plot of unadjusted P-values from meta-analysis of EWAS results for FIB-4 score. The red line indicates the unadjusted P-value that corresponds to a threshold for FDR significance at Q < 0.05. DNA methylation was not significantly associated with FIB-4 score for any CpG site after a more restrictive Bonferroni correction to an overall a = 0.05.

**Supplementary Figure 8:**

Distribution of unadjusted P-values from meta-analysis of EWAS results from the EPIC and 450K cohorts for APRI score. (A) Quantile-quantile plot of unadjusted P-values from meta-analysis of EWAS results for APRI score. The global inflation factor was 1.08, so no further adjustment for global inflation was performed. Red lines represent a perfect 1:1 association with a 95% confidence interval. (B) Manhattan plot of unadjusted P-values from meta-analysis of EWAS results for APRI score. The red line indicates the unadjusted P-value that corresponds to a threshold for FDR significance at Q < 0.05. DNA methylation was not significantly associated with APRI score for any CpG site after a more restrictive Bonferroni correction to an overall a = 0.05.
