# Supplementary figures and images for: Epigenome-wide association study of biomarkers of liver function identifies albumin-associated DNA methylation sites among male veterans with HIV
Source: Front Genet. 2022 Oct 11;13:1020871. doi: 10.3389/fgene.2022.1020871 (PMC9592923; doi:10.3389/fgene.2022.1020871)

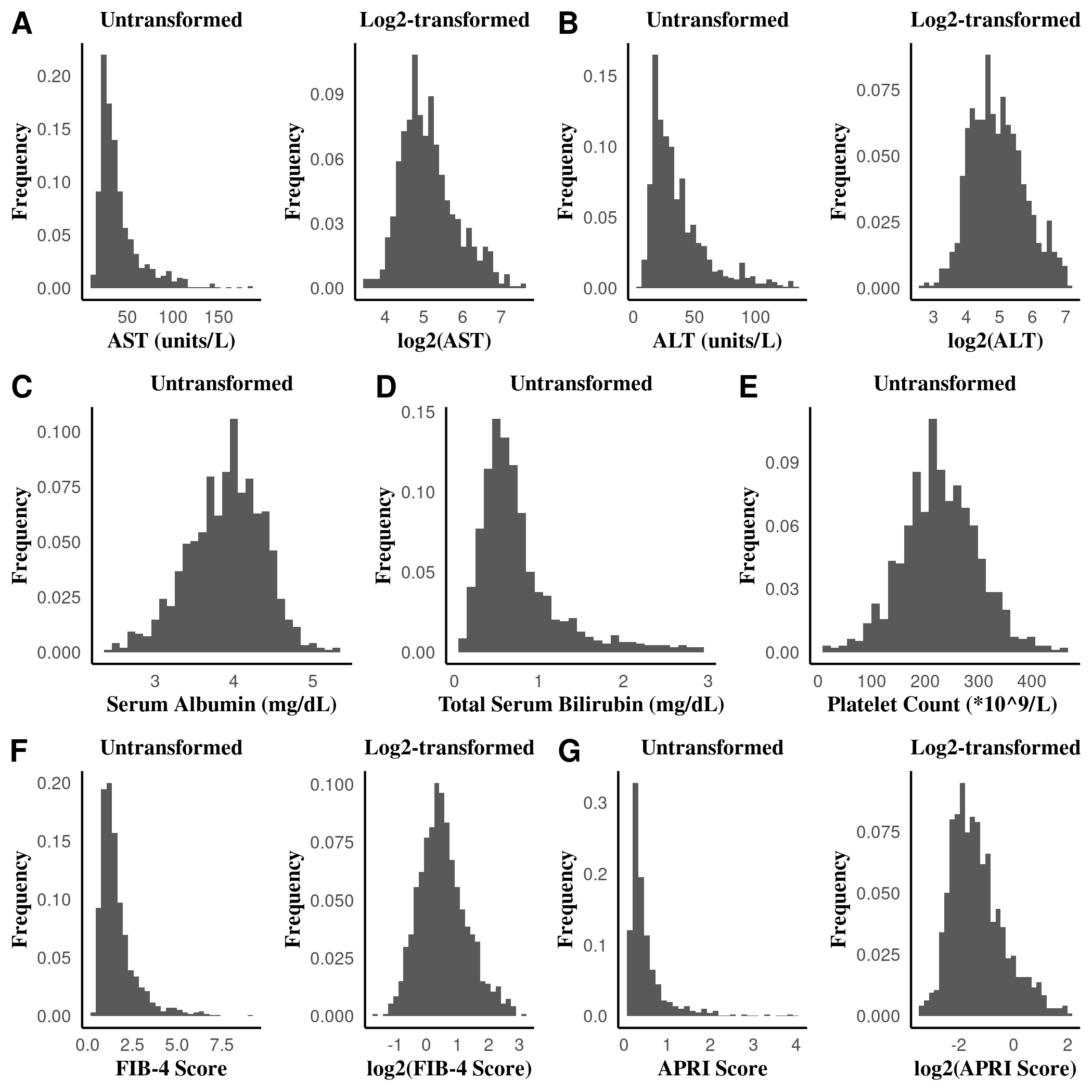

Supplement: Supplementary file 3 [file Image1.JPEG]
