# Supplementary material for: Epigenome-wide association study of biomarkers of liver function identifies albumin-associated DNA methylation sites among male veterans with HIV
Source: Front Genet. 2022 Oct 11;13:1020871. doi: 10.3389/fgene.2022.1020871 (PMC9592923; doi:10.3389/fgene.2022.1020871)
Supplement: Supplementary file 5 [file DataSheet1.PDF]

Supplementary Figure 1:
Distributions of values for selected biomarkers of liver function among the entire cohort. Only biomarkers with highly right-skewed distributions were log-transformed to produce more normal distributions that allow for inference from linear modelling. For this figure, log2 transformation was used for easy interpretation. For EWAS, a natural log transformation was applied when needed. A) Distribution of AST before and after log transformation. B) Distribution of ALT before and after log transformation. C) Distributions of ALB without log transformation. D) Distributions of ALB without log transformation. E) Distributions of ALB without log transformation. F) Distribution of FIB-4 scores before and after log transformation. G) Distribution of APRI scores before and after log transformation. Abbreviations: AST, aspartate aminotransferase level (units/L); ALT, alanine aminotransferase level (units/L); ALB, serum albumin level (mg/dL); TBILI, total bilirubin level (mg/dL); PLT, platelet count (cells/mL); FIB-4, FIB-4 score; APRI, APRI score.

Supplementary Figure 2:
Distribution of unadjusted P-values from meta-analysis of EWAS results from the EPIC and 450K cohorts for AST, ALT and Total Bilirubin. (A, C, E) Quantile-quantile plot of unadjusted P-values from meta-analysis of EWAS results for AST, ALT and Total Bilirubin. The global inflation factor was 1.04, 1.18, and 1.04 respectively, so no further adjustment for global inflation was performed. Red lines represent a perfect 1:1 association with a 95% confidence interval. (B, D, F) Manhattan plot of unadjusted P-values from meta-analysis of EWAS results for AST, ALT and Total Bilirubin. The red line indicates the unadjusted P-value that corresponds to a threshold for FDR significance at $Q < 0.05$.

Supplementary Figure 3:
Distribution of unadjusted P-values from meta-analysis of EWAS results from the EPIC and 450K cohorts for platelet count, FIB-4 score and APRI score. (A, C, E) Quantile-quantile plot of unadjusted P-values from meta-analysis of EWAS results for platelet count, FIB-4 score and APRI score. The global inflation factor was 1.02, 0.959 and 1.08 respectively, so no further adjustment for global inflation was performed. Red lines represent a perfect 1:1 association with a 95% confidence interval. (B, D, E) Manhattan plot of unadjusted P-values from meta-analysis of EWAS results for platelet count, FIB-4 score and APRI score. The red line indicates the unadjusted P-value that corresponds to a threshold for FDR significance at $Q < 0.05$.
